# Oxandrolone Efficacy in Wound Healing in Burned and Decubitus Ulcer Patients: A Systematic Review

**DOI:** 10.7759/cureus.28079

**Published:** 2022-08-16

**Authors:** Ana Paula C Jalkh, Aziza K Eastmond, Chaitra Shetty, Syed Muhammad Hannan Ali Rizvi, Joudi Sharaf, Kerry-Ann D Williams, Maha Tariq, Maitri V Acharekar, Sara Elena Guerrero Saldivia, Sumedha N Unnikrishnan, Yeny Y Chavarria, Adebisi O Akindele, Pousette Hamid

**Affiliations:** 1 Research, California Institute of Behavioral Neurosciences & Psychology, Fairfield, USA

**Keywords:** anabolic steroids, anavar, oxandrin, pressure ulcer, wound healing, decubitus ulcer, burn, decubitus ulcer healing, burn healing, oxandrolone

## Abstract

Wounds with delayed or impaired healing represent a considerable challenge in medical practice. These patients develop a sustained hypermetabolic and catabolic state, directly impacting the wound healing process. The use of oxandrolone has been studied to control this metabolic imbalance and protect lean body mass as a beneficial resource in wound healing. This systematic review aims to analyze previously conducted randomized controlled trials to evaluate the evidence of the applicability of oxandrolone therapy. We compared its use in adult patients with burns and adult patients with pressure ulcers in terms of wound healing and healing time of the skin graft donor site in days. The digital searches were done from March 23-28, 2022, within the databases: Google Scholar, PubMed/MEDLINE, and EBSCO (Elton B. Stephens Company). Data from six studies were analyzed and included in this review. Analysis of the available data demonstrated a significant advantage in skin healing using oxandrolone in adult burn patients as an adjunct. For adult patients with pressure ulcers, the drug showed no benefit on wound healing and skin graft site healing. Importantly, we found only one study evaluating the use of oxandrolone in patients with decubitus ulcers that met our eligibility criteria, and the certainty of the evidence was low. Thus, further prospective randomized studies with larger samples and standard wound care protocols are needed to produce more solid results, allowing more definitive conclusions to be made on this theme.

## Introduction and background

The annual occurrence of burns in the United States (US) is 1.2 million people. Approximately 60,000 patients will require hospital admission. And regardless of medical care, around 5,000 people die yearly from burns or complications [1]. These numbers can be three to five times higher in developing countries [2]. On the other hand, the annual incidence of pressure ulcers in the US is about one to three million individuals. It represents a government's yearly fee of US$1.6 billion [3]. Wounds from any underlying reason impair sufferers' ability to move, self-care, and conduct everyday living activities. All of those will generate ache and discomfort, negatively influencing patients' and their families' well-being [4,5,6].

In patients with persistent wounds and burns, a hypermetabolic and catabolic state is generated. This abnormal metabolic response increases nutritional needs and is a trigger for the installation of protein-energy malnutrition. Protein-energy malnutrition and activation of the stress response to injury are complicating elements for restoration. Thus, the control of catabolism by using the exogenous replacement of anabolic hormones and the good enough consumption of proteins and micronutrients presents itself as a feasible solution for the protection of lean mass and a helpful resource in wound healing [7,8,9,10,11].

Oxandrolone is a synthetic 17-alpha-methyl derivative of testosterone. It has 10 times more anabolic activity than testosterone and only 10% of its androgenic adverse effects. Time to reach the top concentration, the maximum plasma concentration, and bioavailability no longer modify with age. Therefore, it is considered a safe profile for children and the elderly. Oxandrolone appears to be able to stimulate the expression of androgen receptors in skeletal muscles and make protein synthesis more efficient. It is positively impacting the conservation of lean body mass. Thus, the US Food and Drug Administration (FDA) authorizes its use as an adjuvant therapy to regain body weight after extensive surgery, chronic infections, and severe trauma. The drug also combats the catabolism generated by cortisol through competitive inhibition of the glucocorticoid receptor. In this way, the FDA also authorizes its use to prevent protein catabolism associated with chronic corticosteroid use and to reduce osteoporotic bone pain [7,8,10,12].

There is a widespread growth in anabolic activity with the usage of anabolic steroids. The growing evidence of direct stimulation of all phases of wound healing is of great clinical interest. However, the particular mechanism is not clear. Studies show androgen receptors in high concentration in skin myocytes and fibroblasts. Also, a few epithelial cell populations appear to accommodate these receptors. Once these androgen receptors are activated, there is a local release of growth factors. Growth factors attract cells to the wound, drive their proliferation, and lead to extracellular matrix deposition. Other studies point to oxandrolone as a stimulator of procollagen messenger ribonucleic acid (RNA) expression in fibroblasts. In such a way, increasing collagen synthesis and deposition in the healing wound [7,8,10,11,12,13,14,15,16].

This systematic review aims to appraise the evidence of the applicability of oxandrolone therapy in adult patients with burns compared to adult patients with pressure ulcers in terms of wound healing and healing time of the skin graft donor site in days.

## Review

Methods

This systematic review is centered on clinical, observer-noted final results in randomized controlled trials (RCTs). It was carried out under the Preferred Reporting Items for Systematic Reviews and Meta-Analyses (PRISMA) guidelines [17]. 

Databases

The digital searches were done from March 23-28, 2022, within the databases: Google Scholar, PubMed/MEDLINE, and EBSCO (Elton B. Stephens Company). We adjusted the search strategy for each database to obtain the most significant results. In Google Scholar and EBSCO, we defined the search into the first 10 pages for each keyword "oxandrolone AND burn healing" and "oxandrolone AND decubitus ulcers." Limiting the search in these databases allowed us to balance workload and the expectation of choosing sufficient studies achieving inclusion criteria. On PubMed, Medical Subject Headings (MeSH) search strategy was used: ("Oxandrolone/therapeutic use"[Majr]) AND "Burns"[Majr]; ("Oxandrolone/therapeutic use"[Majr]) AND "Pressure Ulcer"[Majr]; ("Oxandrolone/therapeutic use"[Majr]) AND "Wound Healing"[Majr].

Eligibility Criteria

Types of studies: We combined full-text RCTs. We excluded guidelines, conference proceedings, abstracts, case reports, literature reviews, systematic reviews, editorials, and book chapters. We also excluded non-English language publications and studies not involving human subjects. Studies where we could not have access to the full text, we also excluded.

Types of participants: We included studies recording outcomes from adult patients of any age with a cutaneous burn of any kind or size, determined by clinician evaluation, which required treatment in any healthcare facility. We also included studies recording outcomes from adult patients of any age, with any baseline disease, with a decubitus ulcer of any size or depth, in any localization, determined by clinician evaluation. We excluded studies with children population.

Type of interventions: RCTs that compared burn or decubitus ulcer patients taking oxandrolone with placebo controls to assess the efficacy of oxandrolone in wound healing and donor-site healing time were included.

Types of outcomes: Defined as the grade of re-epithelization of the wound or donor-site healing time after using oxandrolone. Trials assessing wound or donor-site healing time were only included if the data were observer-reported. This outcome should be compared with placebos to demonstrate the efficacy of this drug usage related to wound healing.

Studies Selection Process and Data Extraction

All review authors underwent online conferences to make identical knowledge of this review's objective and the eligibility criteria. In the initial searches, duplicates were removed manually, and all titles were unified. Two authors (A.P.C.J and A.K.E.) individually evaluated titles and abstracts. In case of disagreements, a third reviewer (C.S.) was reached to decide the eligibility. Two reviewers (A.P.C.J and A.K.E.) then read the full-text content articles in their entirety to evaluate for eligibility, with accords on inclusion and exclusion criteria. From a total of 2,759 articles initially identified, six RCTs were judged to be eligible for incorporation in our review. We excerpted the particular characteristics of each qualified study and added them to a table in Microsoft Excel (Microsoft Corporation, Redmond, Washington, US) to facilitate the analysis. We pulled the following data from each study: authors and year of publication, country of origin, study design, study population characteristics, sample size, drug regimen, time-to-treatment, length of treatment, follow-up time, and outcomes reporting p-values.

Quality Appraisal of the Trials

We assessed the chance of bias in selected randomized clinical trials using the Cochrane risk of bias tool [18]. Six of the seven domains were evaluated: random sequence generation, allocation concealment, blinding of participants and professionals, blinding of outcome assessors, incomplete outcomes, and selective outcome reporting. Each domain was counted as "low," "high," or "unclear." Therefore, studies were judged to have a "low," "moderate," or "high" chance of bias consistent with the rating system. All studies included were classified as having a low risk of bias. See Table 1 for clarification of the risk of bias assessment across the research.

**Table 1:** Assessment of clinical trials using Cochrane risk of bias tool for included studies. RCT: randomized controlled trial Source: The Cochrane Collaboration’s tool for assessing the risk of bias in randomized trials [18].

RCT	Random Sequence Generation	Allocation Concealment	Blinding of Participants and Professionals	Blinding of Outcome Assessors	Incomplete Outcomes	Selective Outcome Reporting
Demling, 1999 [14]	Low risk	Unclear	Low risk	Low risk	Low risk	Low risk
Demling and Orgill, 2000 [19]	Low risk	Low risk	Low risk	Low risk	Low risk	Low risk
Demling and DeSanti, 2003 [20]	Low risk	Unclear	Low risk	Low risk	Low risk	Low risk
Bauman et al., 2013 [21]	Low risk	Low risk	Low risk	Low risk	Low risk	Low risk
Demling and DeSanti, 2002 [22]	Low risk	Unclear	Low risk	Low risk	Low risk	Low risk
Demling and DeSanti, 2001 [23]	Low risk	Unclear	Low risk	Low risk	Low risk	Low risk

Results

The search strategy for identifying relevant studies within the databases Google Scholar, PubMed/Medline, and EBSCO was completed as described in the Methods section. A total of 2,759 records were identified and analyzed as follows.

For the term "oxandrolone AND burn healing" in Google Scholar, we located 1,440 citations. We screened the title and abstract in the first 10 pages, which yielded 99 citations. Of these, we excluded 19 studies in children, five in animals, and one was a duplicate study. Forty-two citations were excepted because the title and abstract did not match the objectives of this systematic review. Nineteen were excluded for being editorials, thesis, expert opinions, recommendations, literature reviews, systematic reviews, or book chapters. For the terms "oxandrolone AND decubitus ulcers" in Google Scholar, we found 821 citations. In the first 10 pages, we went through 89 articles. Of these, we excluded six duplicates, two animal studies, and three articles lacking extractable data. Fifty-two citations were excepted because the title and abstract did not match the objectives of this systematic review. Twenty-three were excluded for being non-RCT studies.

On PubMed, MeSH search strategy was used. For the term ("Oxandrolone/therapeutic use"[Majr]) AND "Burns"[Majr], we found 29 citations. When we applied an RCT as a filter, we found 16 results in the English language. We excluded 11 studies for being in the children population and two studies due to no match between the title and the abstract. The final number turned to three studies which all were duplicates. For the terms ("Oxandrolone/therapeutic use"[Majr]) AND "Pressure Ulcer"[Majr], we found seven articles. It became a duplicate result when we applied RCTs and the English language as filters. For the terms ("Oxandrolone/therapeutic use"[Majr]) AND "Wound Healing"[Majr], we found eight articles. When we applied an RCT as a filter, it turned to three results in the English Language. All were duplicates.

On EBSCO, we searched for the term "oxandrolone AND burn healing" and found 326 citations. When we applied academic journals and the English language as filters, we turned to 182 sources. We limited our search to the first 10 pages, found 100 results, and screened the title and abstract. We excluded six studies in children, 11 studies in animals, and 25 duplicates. Fifty-two citations were excepted because the title and abstract did not match the objectives of this systematic review. And six for being non-RCT studies. When we searched the term "oxandrolone and decubitus ulcers," we found 128 citations. When we applied academic journals and the English Language as filters, we turned to 27 duplicated citations.

Additionally, we succeeded in a manual search using reference screening and citation tracking of included studies and redeemed one article, Demling and DeSanti, 2001 [23], that satisfied the eligibility criteria.

**Figure 1:**
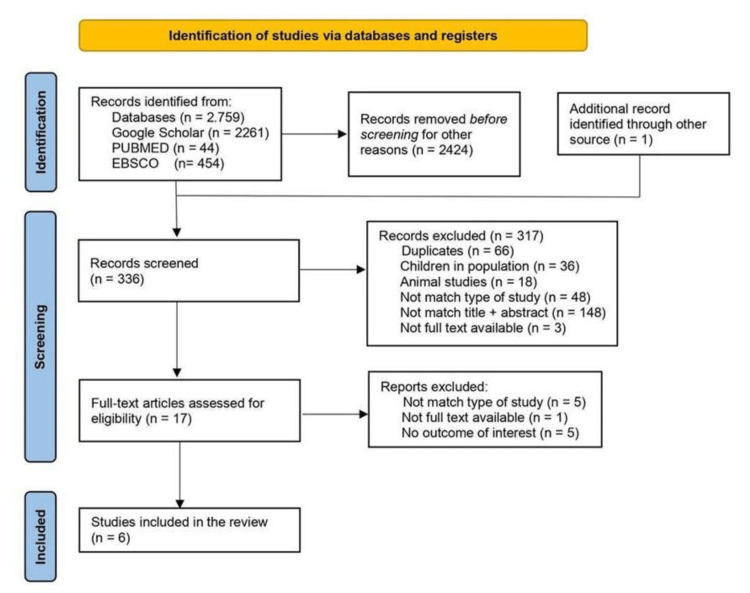
PRISMA flow diagram. PRISMA: Preferred Reporting Items for Systematic Reviews and Meta-Analyses.

Finally, six articles were included in our review. Figure 1 represents the PRISMA flow diagram delineating the study identification, selection, and inclusion process used in the present review.

Baseline Characteristics of Included Studies

The characteristics of the included studies are shown in Table 2.

**Table 2:** Characteristics of studies included in the review. USA: United States of America, TBS: total body surface, HGH: human growth hormone, Ox: oxandrolone, mg/kg/day: milligram per kilogram per day, mg/day: milligram per day, mg: milligram, SCI: spinal cord injury, PU: pressure ulcer, bid: two times a day, N/A: not applicable, HC: hydrocortisone, vs.: versus, p: p-value, y: years.

Authors, Year	Country	Study Design	Study Population Characteristics	Sample Size		Drug Regimen	Time-to-treatment	Length of Treatment	Follow-up Time	Efficacy (Oxandrolone vs. Control)
				Treatment	Placebo					
Demling, 1999 [14]	USA	Randomized prospective	Burned ≥ 50% TBS or ≥ 25% TBS with major comorbidities	20 HGH	24	HGH 0.1mg/kg/day	7-10 days post-burn	Until healed and transferred to a rehabilitation center	12 weeks	Faster healing time: 4 days less with Ox (p< 0.05)
				16 Ox		Ox 20mg/day				
Demling and Orgill, 2000 [19]	USA	Randomized double-blinded	Burned 40-70% TBS	11	9	Ox 20mg/day	2-4 days post-burn	24 - 42 days	N/A	Faster healing time: 4 days less with Ox (p< 0.05)
Demling and DeSanti, 2003 [20]	USA	Randomized prospective	˃ 65 years burned 10-29% TBS	26	24	Ox 10mg bid	N/A	Until discharge or transfer to a rehabilitation center	N/A	↓ time to heal standard donor site (30% less with Ox) (p< 0.05)
Bauman et al., 2013 [21]	USA	Parallel group, randomized	SCI with stage 3 or 4 pressure ulcer	108	104	Ox 10mg bid	After 26-30 days (observation period)	Until PU healed or 24 weeks	8 weeks	No significant difference between groups (p< 0.40)
Demling and DeSanti, 2002 [22]	USA	Randomized prospective	Burned ≤ 55% TBS	15 (group 18-45y)	14 (group 18-45y)	Ox 10mg bid	Recovery phase post-injury (resolution of the "stress response" to injury)	4 weeks or restoration of pre-injury function and weight	N/A	Faster healing time: 4-5 days less with Ox (p< 0.05)
				11 (group ≥ 60y)	11 (group ≥ 60y)					
Demling and DeSanti, 2001 [23]	USA	Randomized prospective	Burned with 24-42% TBS	12 burned	15 burned	Burns: Ox 20mg/day + HC 100 to 200mg/day	2 days post-burn unit admission	3-4 weeks	N/A	Faster healing time: 7-8 days less with Ox (p< 0.05)
			Patients with skin slough 27-57% TBS	10 skin slough patients	10 skin slough patients	Skin slough: Ox 20mg/day + HC 115 to 265mg/day				

Discussion

Wounds with delayed or impaired healing keep patients in a prolonged catabolic state and more prone to infections. At the same time, pain, mobility difficulties, and unsightly scars impact patients' quality of life. Therefore, these wounds represent fantastic defiance of clinical exercise even today [24,25,26].

This systematic review is intended to help us clarify whether the use of oxandrolone accelerates wound healing and donor-healing sites in adult burn patients when compared to adult patients with pressure ulcers.

From this angle, oxandrolone becomes an exciting and cost-effective adjuvant drug. It is administered orally and is considered to have a safe profile even at the extremes of age. Likewise, the drug showed in medical literature reasonable and secure outcomes in pediatric burn victims at 12 months and five-year follow-up after hospital release [10,27,28,29]. It has additionally demonstrated safety in elderly patients over 65 years of age. In this subpopulation, even when creatinine clearance turned to less than 25% of average for age, it was shown to be safe with a dose adjusted to 10 mg daily [20]. In addition, it has an anti-catabolic effect and appears to stimulate all stages of wound healing [7,8,30,31,32,33]. The most common side effect described is an elevation of liver enzymes and a decrease in high-density lipoprotein (HDL) cholesterol levels that regularize after drug withdrawal and do not contraindicate its use or reinstitution after normality [32,34]. It is worth mentioning its contraindication in patients with elevated prostate screening antigen (PSA) or patients with prostate or breast carcinoma history [35].

Our review compiled six studies. Five of these, Demling, 1999 [14], Demling and Orgill, 2000 [19], Demling and DeSanti, 2003 [20], Demling and DeSanti, 2002 [22], and Demling and DeSanti, 2001 [23], included burn patients evaluating the healing time in days of the skin graft donor site using oxandrolone comparing with a placebo group. And only one of these studies, Bauman et al., 2013 [21], evaluated the healing time of stage III and IV chronic pressure ulcers in adult patients with spinal cord injury compared with a placebo group.

A total of 208 burn patients were evaluated, 101 using oxandrolone and 107 controls. All selected patients were considered high-risk patients and with a sufficient burn size to produce a severe catabolic state. Most patients in these studies had nutritional support. Demling, 1999 [14], Demling and Orgill, 2000 [19], and Demling and DeSanti, 2002 [22] started nutritional support on the second day after the burn injury. In the study by Demling and DeSanti, 2003 [20], despite explaining nutritional support in its methodology, it was unclear when this support began. Severe burn injury caused marked catabolism resulting in loss of lean body mass and decreased protein stores, negatively impacting wound healing. To counterbalance this negative outcome, the optimization of protein intake is necessary for the effectiveness of the anabolic agent [14,16,20]. Demling and DeSanti, 2001 [23] did not report nutritional support despite having a positive outcome regarding wound healing in burn patients with skin slough disorders who received high-dose corticosteroids. The efficiency of oxandrolone in wound healing has been demonstrated during corticosteroid therapy, but its action is unclear. It is essential to point out that oxandrolone would not be interesting clinically if it blunted corticosteroids' anti-inflammatory effects [12].

In the studies by Demling, 1999 [14], Demling and Orgill, 2000 [19], Demling and DeSanti, 2003 [20], and Demling and DeSanti, 2001 [24], the anabolic steroid was started between two and 10 days after the injury. In the study by Demling and DeSanti, 2002 [22], its use started in the post-catabolic or recovery phase. Donor sites were monitored for the rate of healing obtained from an unburned area on the arm or leg and assessed by a trained nursing assessor. In addition, early sequential excision and grafting treated all deep burns. These procedures block inflammatory stimulation and minimize hypertrophic scarring [36,37,38]. In all studies involving burned patients using oxandrolone, there was a significant increase in the rate of healing of the donor area and a faster re-epithelialization time compared to placebo (p < 0.05).

The only study that evaluated oxandrolone as a possible adjunct to pressure ulcer healing treatment was by Bauman et al., 2003 [21]. It's a multicenter study designed to test the effectiveness of oxandrolone in accelerating the healing of difficult-to-heal subdermal pressure ulcers compared to the placebo group. These ulcers were defined by an area reduction of ≤ 30% or worsening when observed for 28 (+- 2 days) despite standard treatments. In addition, as a secondary outcome, the percentage of patients with healed ulcers that remained closed after eight weeks of treatment was evaluated. Nutritional support was not provided in the study, and the patients' wounds were reviewed by a site coordinator weekly for 24 weeks. In both groups (oxandrolone and controls), similar numbers of pressure ulcers healed (about 25% to 30%) and remained healed eight weeks after treatment (about 15%) [21]. It is worth observing that the trial stopped early due to the authors' futility analysis (interim analysis). The withdrawal rate also was relatively high and similar between the groups. The study also discussed limitations regarding the uniformity of primary clinical care for patients with pressure ulcers across participating centers. It stressed that the degree of bladder or bowel incontinence was not systematically captured or controlled. All these factors would be highly relevant to the measurements of the final results. Thus, its conclusion section does not rule out the possibility of oxandrolone being beneficial in accelerating wound healing. In other words, the certainty of the evidence was low.

This is the first systematic review focused exclusively on comparing the efficacy of oxandrolone in wound healing in burn and pressure ulcer adult patients. Double-blinded randomized trials agree that the monitored use of oxandrolone in adult burn patients should be considered beneficial as an adjunct with positive effects on skin healing.

In the case of oxandrolone use in patients with pressure ulcers, we found only one multicentric study focusing on wound healing and with unpromising results in facilitating wound healing or keeping a healed wound closed. Based on this literature review, there is no evidence of benefit for suggesting the use of oxandrolone in the treatment of pressure sores.

Limitations

As a limitation of our review, we found few studies focused on the central question of this study. We only included six studies that met our eligibility criteria. The sample size was small despite the outcome results being considered statistically significant. Additionally, despite being multicenter, the only study we found on oxandrolone in patients with pressure ulcers did not perform wound care standardization between centers. The study was completed before the planned recruitment, and the withdrawal rate was relatively high.

## Conclusions

This systematic review suggests oxandrolone accelerates wound healing at skin graft donor sites and wound re-epithelialization in adult burn patients. Monitoring the level of liver enzymes is necessary. Regarding the effectiveness of oxandrolone in treating patients with difficult-to-heal pressure ulcers, the study showed no evidence of facilitation. However, this study itself did not rule out a possible benefit of the drug on wound healing. The certainty of the evidence was low. Therefore, the need for further studies, if possible, prospective, randomized controlled, multicenter, with greater sample size remains. There is also a requirement for defined protocols for nutritional support and uniform wound care among participating centers.
